# Adrenergic β2 Receptor Activation Stimulates Anti-Inflammatory Properties of Dendritic Cells *In Vitro*

**DOI:** 10.1371/journal.pone.0085086

**Published:** 2014-01-22

**Authors:** Laurens E. Nijhuis, Brenda J. Olivier, Shobit Dhawan, Francisca W. Hilbers, Louis Boon, Monika C. Wolkers, Janneke N. Samsom, Wouter J. de Jonge

**Affiliations:** 1 Tytgat Institute for Gastro-Intestinal and Liver Research, Academic Medical Center, Amsterdam, The Netherlands; 2 Bioceros B.V., Yalelaan 46, Utrecht, The Netherlands; 3 Laboratory of Pediatric Gastroenterology, Erasmus University Medical Center, Rotterdam, The Netherlands; 4 Sanquin Research/Landsteiner Laboratory, Department of Hematopoiesis, Amsterdam, The Netherlands; University of Melbourne, Australia

## Abstract

Vagal nerve efferent activation has been shown to ameliorate the course of many inflammatory disease states. This neuro-modulatory effect has been suggested to rest on acetylcholine receptor (AChR) activation on tissue macrophages or dendritic cells (DCs). In more recent studies, vagal anti-inflammatory activity was shown involve adrenergic, splenic, pathways. Here we provide evidence that the adrenergic, rather than cholinergic, receptor activation on bone marrow derived DCs results in enhanced endocytosis uptake, enhanced IL-10 production but a decreased IL-6, IL-12p70 and IL-23 production. In antigen specific T cell stimulation assays, adrenergic β2 receptor activation on bone marrow DCs led to an enhanced potential to induce Foxp3 positive suppressive Treg cells. These effects were independent of IL10-R activation, TGFβ release, or retinoic acid (RA) secretion. Hence, adrenergic receptor β2 activation modulates DC function resulting in skewing towards anti-inflammatory T cell phenotypes.

## Introduction

It has become clear that interaction of the nervous system with antigen presenting immune cells contributes to the homeostatic state of the body [1], [2]. During inflammation, reflex activation of the autonomous nervous system via afferent fibers can lead to activation of efferent signals that can modulate local inflammatory responses via the release of sympathetic and parasympathetic neurotransmitters and neuropeptides [3]. Vagal signaling has been shown to ameliorate disease in a range of inflammatory disease models such as experimental colitis [4] and post-operative ileus [5], an effect that is suggested to depend on acetylcholine receptor (AChR) activation on tissue macrophages [6]. The latter effect may be mediated via direct release of acetylcholine (ACh) and interaction with cholinergic receptors [2], [5], [7], or be mediated via postganglionic adrenergic activity [8]–[10]. However, para- and sympathetic systems work in tandem and stimulation of vagus nerve output may well affect sympathetic activity and catecholamine release as shown earlier [1], [9].

However, a variety of neurotransmitters in addition to ACh, such as catecholamines (nor)epinephrine and several neuropeptides, can influence the function of myeloid immune cells such as macrophages but also dendritic cells (DCs) [11], [12], an effect that may have been largely ignored in earlier models. DCs are specialized antigen presenting cells (APCs) with the unique ability to initiate and polarize adaptive immune responses. DCs act as innate immune sensors and capture antigens via endocytosis. Murine bone marrow derived dendritic cells (BMDC) express nicotinic AChR, muscarinic AChR, adrenergic receptors (AR) and several peptidergic receptors like the vasointestinal peptide receptors VPAC1 and 2. Activation of these receptors and subsequent modulation of DC function has been studied earlier [11]–[13], but conclusions about the net effect have been inconsistent.

To clarify the role of adrenergic and cholinergic receptor signaling on the modulation of DC function, we compared the effect of the (para)sympathetic agonists ACh, nicotine, and epinephrine, on various DC functions such as endocytosis, maturation, cytokine production and assess the ability of these DC to induce/drive T helper (Th) cell differentiation. We establish a potential of sympathetic neurotransmitter NE to stimulate IL-10 secretion whilst reducing IL12-p70 production. This effect corresponded with a Th2 and regulatory T-cell (Treg) skewing potential of BMDCs pre-exposed to AR-β2 agonists. For this reason, AR-β2 agonists may be considered as anti-inflammatory agents in inflammatory diseases in which inflammatory DC activity plays a causative role.

## Materials and Methods

### Mice

C57BL/6 inbred mice were purchased from Charles River (Maastricht, The Netherlands). All mice were female, 2–3 months old and maintained in our animal facility under standard 12-h photoperiod, at 21±1°C, with food and water *ad libitum*. Female mice were chosen to avoid fighting behavior prior to cell harvesting. Before bone marrow cell isolation, mice were acclimated at housing facility for 7 days to eliminate shipping stress. Female and male OT-II transgenic mice were bred in the AMC animal facility. The animal experiments committee of the University of Amsterdam approved all experiments described in this study.

### Bone marrow-derived DCs

Murine BMDCs from femur and tibia of C57BL/6 mice were generated as previously described [14]. Cells were cultured in 10 cm petri dishes (Greiner Bio One, Germany) at a concentration of 5×10^6^ cells/10 mL at 37°C, 5% CO2 in complete medium: RPMI 1640 (Gibco, Paisley, UK) containing 10% heat inactivated FCS, 2 mM l-glutamine 100 U/ml Penicillin, 100 µg/ml streptomycin all from (Lonza, Belgium), 50 µM 2-mercaptoethanol (Sigma-Aldrich, Zwijndrecht, The Netherlands), and 20 ng/ml granulocyte/macrophage-colony-stimulating factor (GM-CSF, Peprotech). At day 3, 10 ml of fresh medium was added to each plate supplemented with 10 ng/ml GM-CSF. At day 6 half of culture medium was replaced with fresh medium supplemented with 10 ng/ml GM-CSF. At day 8 non-adherent cells were collected as immature BMDCs. The purity of CD11c+ cells obtained was routinely >90% as assessed by flow cytometry.

### In vitro experiments

Day 8 immature BMDCs were cultured at a concentration of 1×10^6^ cells/ml in complete medium in 24 wells suspension plates. Based on prior dose-finding using maturing dendritic cells, cells were either incubated with 1 µM of acetylcholine, nicotine, epinephrine, salbutamol (all from Sigma-Aldrich) or medium for 20 min. To block AR-β receptors, cells were pre-incubated for 20 min. with 1 µM propranolol (Sigma-Aldrich). Immature BMDCs were stimulated with 100 ng/ml ultra pure LPS (Invivogen, San Diego, CA) for 24 hours to obtain mature BMDCs. Supernatant was collected for cytokine protein quantification by sandwich ELISA for IL-12p70, IL-10, IL-17A and IL-23 (R&D systems, Abingdon, UK). For measurement of co-stimulatory molecule expression, BMDCs were incubated for 30 min. 4°C with allophycocyanin conjugated anti-CD11c (clone HL3) and fluorescein isothiocyanate (FITC) conjugated anti-MHCII (clone AF6-120.1) (BD Biosciences), anti-CD80 FITC (clone 16-10A1), anti-CD86 PE (clone GL1), anti-CD40 FITC (clone HM40-3) (eBiosciences, San Diego, USA), washed twice and positive cells for immunostaining were identified using the flow cytometry analyzer Calibur (BD Biosciences). Further analysis was performed with Flowjo (version 7.6.5.).

For IL-10 and TGF-β blocking experiments we used 1 µg/ml anti-IL-10R (clone 1B1.2), 1 µg/mL anti-TGF-β (clone 1D11) and as an isotype control anti-β-Galactosidase (clone GL113). To block endogenous retinoic acid (RA) production we used 10 µM LE540 (Wako chemicals) and LE135 (Tocris Biosciences). For tyrosine hydroxylase (TH) blockade α-methyl-DL-tyrosine methyl esther hydrochloride (AMPT) (Sigma Aldrich) was used according to manufacturer instructions.

### Real-time RT-PCR

Mature BMDCs were collected and RNA was extracted using TRIpure (Roche, Mannheim, Germany). Genomic DNA was removed using DNase (Promega, Madison WI, USA) and cDNA was synthesized from 1 µg of total RNA using cDNA synthesizing kit (Thermo Scientific, Lithuania). The SYBR green-based real-time PCR technique was used to detect the expression of AR-β1, AR-β2, AR-β3, and TH. The cDNA was diluted 4-fold for the real-time PCR assay. The PCR mixture consists of 1 µl cDNA, 5 µl SYBR-green master mix (Roche) and 1 mM of each primer in a total volume of 10 µl. Real-time PCR was performed using the Lightcycler 480 (Roche) detection system. Cycling conditions used were 95°C for 15 s and 60°C for 1 min, for 40 cycles. Data were analyzed using the ΔΔCt method and results were expressed as fold difference relative to the mean expression of the housekeeping genes β-actin and β2-Macroglobuline (B2M). All primer sequences used are given in Table 1.

**Table 1:** Primer sequences used in this study.

Gene	5′ Primer sequence Forward	5′ Primer sequence Reverse
Adrb1	CACACAGGGTCTCAATGCTG	GATCTGGTCATGGGATTGCT
Adrb2	GAGTGTGCAGGACGCACCCC	CTGTCGTTCCCGTGTGGCCC
TH	AATGGGTTCCCAGGTTCC	AGCAGGATACCAAGCAGGC
β-actin	TGACAGGATGCAGAAGGAGATTAC	AGCCACCGATCCACACAGA
B2M	TGGTCTTTCTGGTGCTTGTCT	ATTTTTTTCCCGTTCTTCAGC

### Endocytosis assay

The endocytosis assay was performed as previously described [15]. Immature BMDCs (1×10^6^/ml) were pre-incubated for 20 minutes with 1 µM of acetylcholine, nicotine, epinephrine or salbutamol in complete medium. The endocytic tracer FITC-Dextran (M.W. 4,000) was added to a final concentration of 1 mg/ml. One plate was incubated at 37°C (control) and the second was incubated on ice and was used as a background control for every time point. Endocytosis was halted at the indicated time points by rapidly cooling the cells on ice followed by 3 washes with ice cold 2% FBS-PBS. Cells were analyzed by flow cytometry. The geometric mean fluorescent intensity difference between 37°C and 4°C was considered as the result of antigen uptake.

### In vitro stimulation of naïve OVA-specific CD4+ T cells

Day 8 immature BMDCs were incubated for 6 hours with Ovalbumin (Grade VI, Sigma-Aldrich) at a concentration of 250 µg/ml. Cells were washed and pre-incubated with vehicle, epinephrine or salbutamol, 1 µM for 20 min, before stimulation with LPS for 24 hours. Spleen cells from OT-II mice were prepared and CD4^+^CD62L^+^ naïve T cells were isolated by magnetic cell sorting (MACS) according to manufacturer's instructions (Miltenyi Biotech).

To examine the potential effect of AR-stimulated BMDCs to alter T cell proliferation, matured BMDCs were washed twice to remove compounds and LPS, and freshly isolated naïve T cells were stained with 5, 6-carboxy-succinimidyl-fluoresceine-ester (CFSE, Molecular Probes, Eugene, Oregon) and co-cultured with BMDCs for 4 days. Proliferation was measured by flow cytometry.

To investigate the effect of AR-stimulated BMDCs on T cell differentiation, matured BMDCs were co-cultured with naïve OT-II T cells for 5 days in 96 wells plates at a BMDC:T cell ratio of 1∶10. Supernatant was collected at day 4 of co-culture for cytokine measurement by ELISA and fresh medium was added. At day 5, T cells were harvested, washed and restimulated with 100 ng/ml phorbol myristate acetate (PMA) and 1 µg/ml ionomycin (both from Sigma-Aldrich). After two hours 10 µg/ml Brefeldin A (Sigma-Aldrich) was added and cells were incubated for 6 hours. Cells were stained for CD4 (eBiosciences) and fixed in 4% paraformaldehyde (Merck, Darmstadt, Germany) permeabilized using 0.5% Saponin (Sigma-Aldrich) for intracellular staining for IL-4, IL-17A (eBioscience) and IFNγ (BD Biosciences) or fixed and permeabilized with Foxp3 staining kit (eBiosciences) for Foxp3 expression according to manufacturer's instructions and characterized by flow cytometry.

### Aldefluor assay

Immature BMDCs were pre-incubated with epinephrine, propranolol followed by epinephrine or vehicle control and activated with LPS for 24 hrs. After maturation cells were harvested and RALDH activity in individual cells was measured using Aldefluor staining kit (StemCell Technologies, Grenoble, Fr), according to the manufacture's protocol with modifications. Briefly, cells were suspended at 10^6^ cells/ml in Aldefluor assay buffer containing activated Aldefluor reagent (1.5 µM) with or without the RALDH inhibitor diethylaminobenzaldehyde (DEAB, 15 µM) were incubated for 40 min at 37°C. The cells were subsequently stained with APC conjugated anti-CD11c (BD Biosciences) in ice-cold Aldefluor assay buffer. Aldefluor reactive cells were detected using a FACSCalibur flow cytometer. Cell viability was determined by flow cytometry with 7AAD exclusion.

### Statistical analysis

Statistical analysis was performed using GraphPad Prism Version 5.0 (GraphPad Software, San Diego, USA). For multi-experimental group analysis, data were subjected to one-way or two-way ANOVA (analysis of variance) followed by post hoc test (Bonferroni and Newman-Keuls) for group differences. All data are expressed as means ± standard error of mean (SEM). The two-tailed level of significance was set at p≤0.05, 0.01, 0.001 for group differences.

## Results

### β2-AR activation modulates cytokine profile of maturing BMDCs

First, we determined to what extent neurotransmitters have the potential to modulate the cytokine profile of maturing BMDCs. To this end, immature BMDCs were treated prior to maturation with LPS with indicated doses of epinephrine, the AR-β2 selective agonist salbutamol, and the AR-β antagonist propranolol. For cholinergic modulation of BMDCs we used ACh and nicotine.

**Figure 1:**
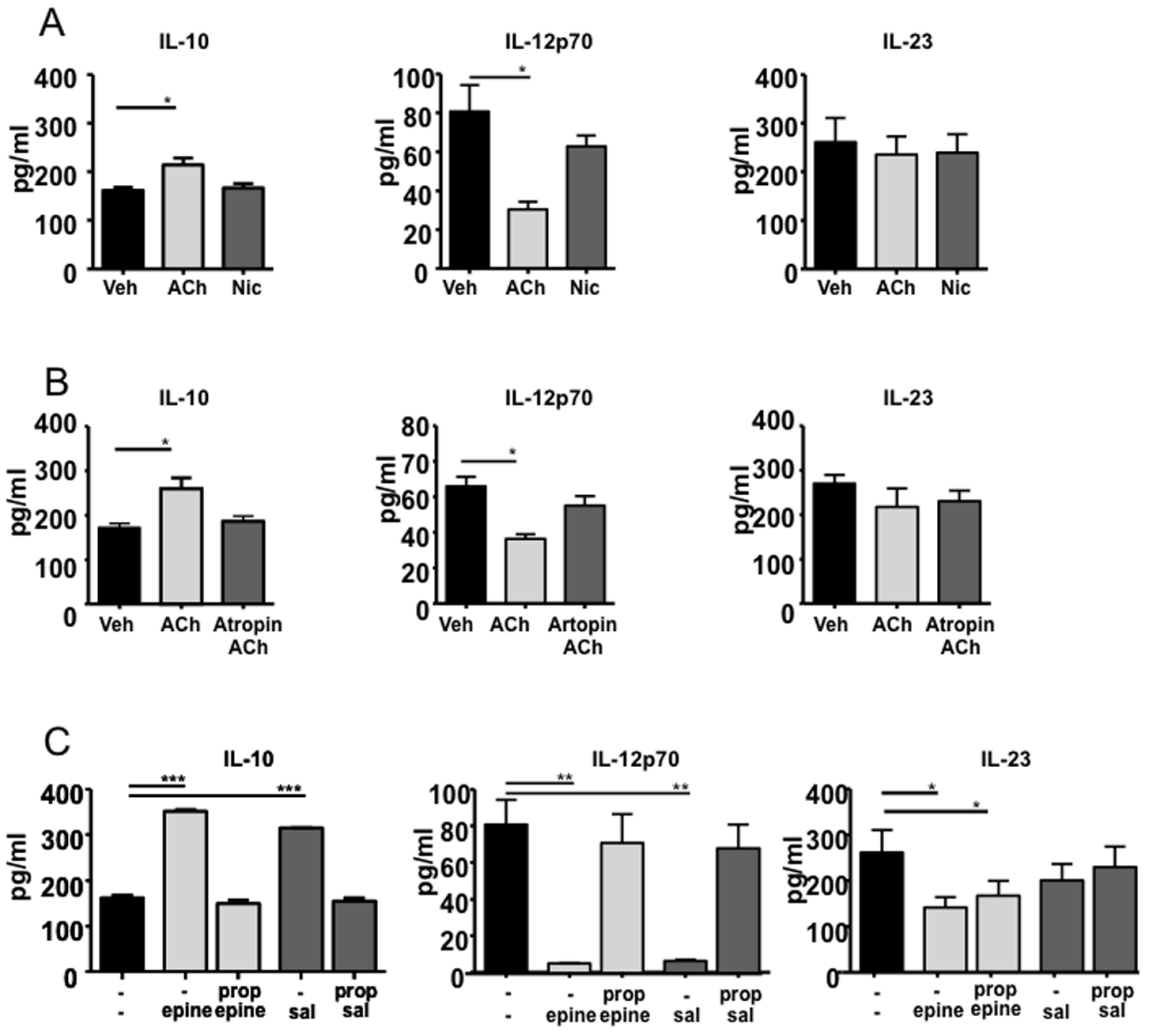
Effect of adrenergic and cholinergic activation on cytokine production in maturing BMDC after 24 Panel A, IL-10, IL-12p70 and IL-23 concentrations in ACh and Nicotine (Nic; 1 µM) pre-treated BMDC. Panel B, IL-10, IL-12p70 and IL-23 concentrations in epinephrine (epine), salbutamol (sal) and/or propranolol (prop) (all at 1 µM) pre-treated BMDC. The values are expressed in pg/ml and represent the mean ± SEM of three independent experiments representative of 5 experiments. * p<0.05, ** p<0.01, ***p<0.001 (ANOVA).

Cholinergic receptor activation using ACh on BMDCs resulted in a significant increase of IL-10, and decreased levels of IL-12p70 (fig. 1A). The effect of ACh was reproduced using muscarine, and not nicotine, and was blocked by atropine (fig. 1A). The latter data demonstrate that muscarinic rather than nicotinic receptors on BMDCs mediate these changes in cytokine release, supporting earlier reports in human DC cultures [12]. We next tested the potential of cholinergic receptor agonists to AR agonists to modulate BMDC cytokine profile. When LPS-induced maturation of BMDCs was performed after pretreatment with epinephrine or the β2-AR selective agonist salbutamol, IL-10 production was significantly increased, whilst IL-12p70 levels were almost completely blocked compared to vehicle. This effect was counteracted by the general AR-β receptor antagonist propranolol (fig. 1A), further confirming the involvement of AR-β2 in this process. Epinephrine additionally decreased production of the pro-inflammatory cytokine IL-23 to similar extent; however this effect was not significantly blocked by propranolol, and no significant decrease was seen using salbutamol. The effect of epinephrine and norepinephrine on BMDCs were highly comparable and suggest the same mode of action of these two catecholamines (data not shown). As the highest effective dose was 1 µM for both cholinergic as well as adrenergic agonists, we conclude that AR-β2 activation was more potent in modulating BMDC cytokine responses.

### Adrenergic modulation of cytokine secretion involves cAMP dependent pathways

**Figure 2:**
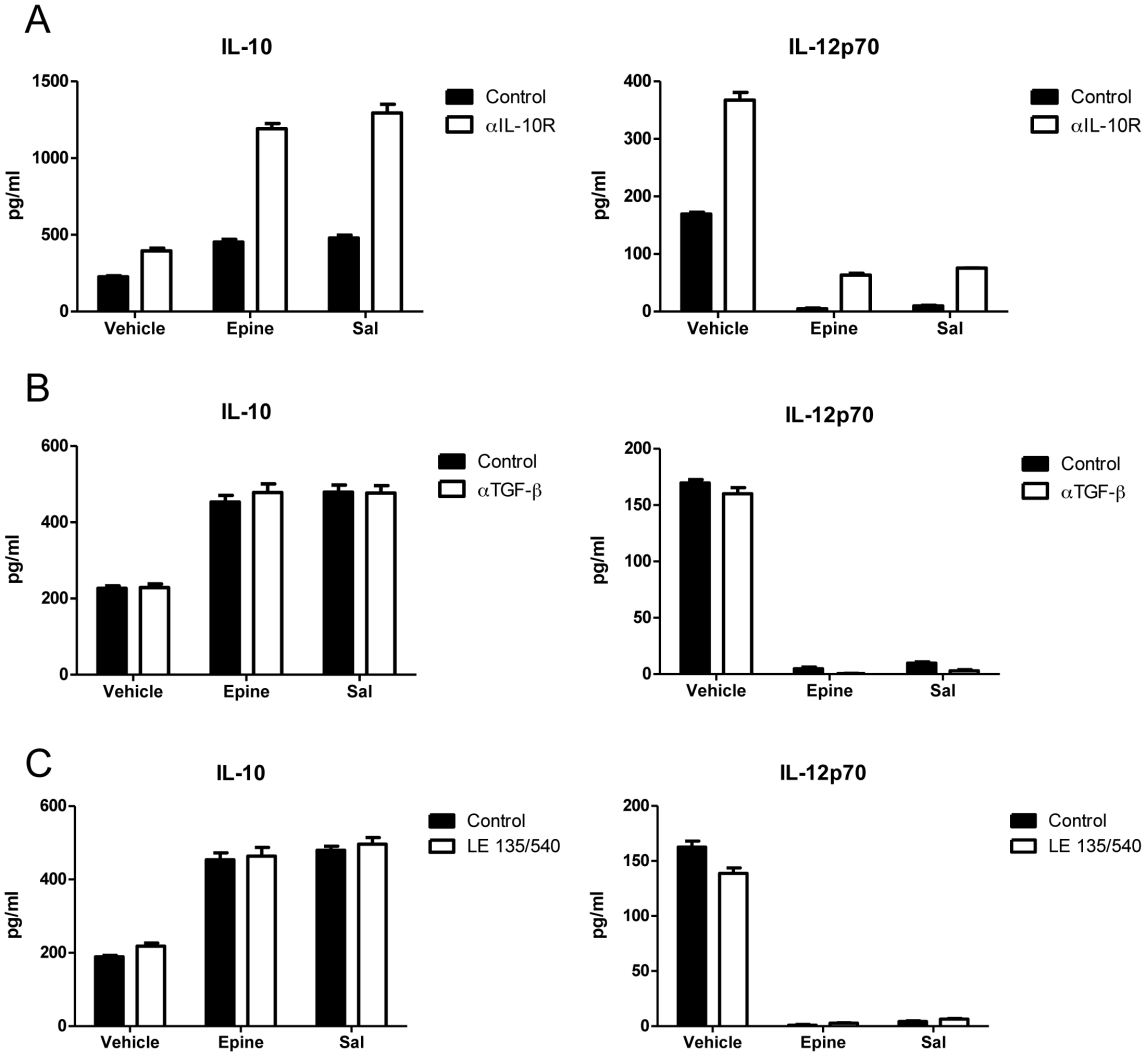
The effect of epinephrine and salbutamol on IL-10 and IL-12p70 production by BMDC in the presence of indicated reagents added. Experiments in the presence of; Panel (A) IL-10R blocking antibody, (B) TGF-β blocking antibody, or (C) blockers of RA signaling in BMDC. Data are expressed in pg/ml and represent the mean ± SEM of two independent experiments using BMDC from three different mice.

We next addressed the mechanism by which AR-β2 regulates BMDC cytokine release. Regulation through AR-β2 could either occur indirectly via the induction of known regulatory molecules such as IL-10 or TGFβ, or be a direct consequence of receptor ligation. First we assessed whether the effects on cytokine production by BMDCs induced by AR-β2 activation depended on the enhanced secretion of IL-10, or activation of TGF-β, or retinoic acid signaling by BMDCs. Thereto, we investigated whether AR-β2 activation was still effective in the presence of blocking antibodies against the receptor for IL-10 or TGF-β, or blockers of retinoic acid (RA) signaling (LE135 and LE540) (fig. 2A–C). Although the blocking antibody against IL-10R increased cytokine responses induced by LPS maturation, the potential of AR-β2 activation to modulate cytokine responses towards IL-10 production in BMDCs was not affected (fig. 2A). Moreover, blocking of TGF-β signaling or the inhibition of retinoic acid receptor signaling did not influence the changes in cytokine profile after epinephrine or salbutamol treatment (fig. 2B and C). To further confirm that the AR-β2 activation in BMDCs is not mediated by altered release of IL-10 or TGF-β receptor signaling but rather involves direct action of the AR-β2 we challenged immature BMDCs with Prostaglandin E2 (PGE2) to mimic the effects of AR-β2 G-protein coupled receptor (GPCR) activation (not shown) through direct induction of cyclic adenosine monophosphate (cAMP) activity. This treatment elicited similar changes in the cytokine profile of maturing BMDCs, demonstrating the direct involvement of AR-β2 in imprinting BMDCs, rather than the autocrine action of secreted cytokines on the maturing BMDCs culture.

**Figure 3:**
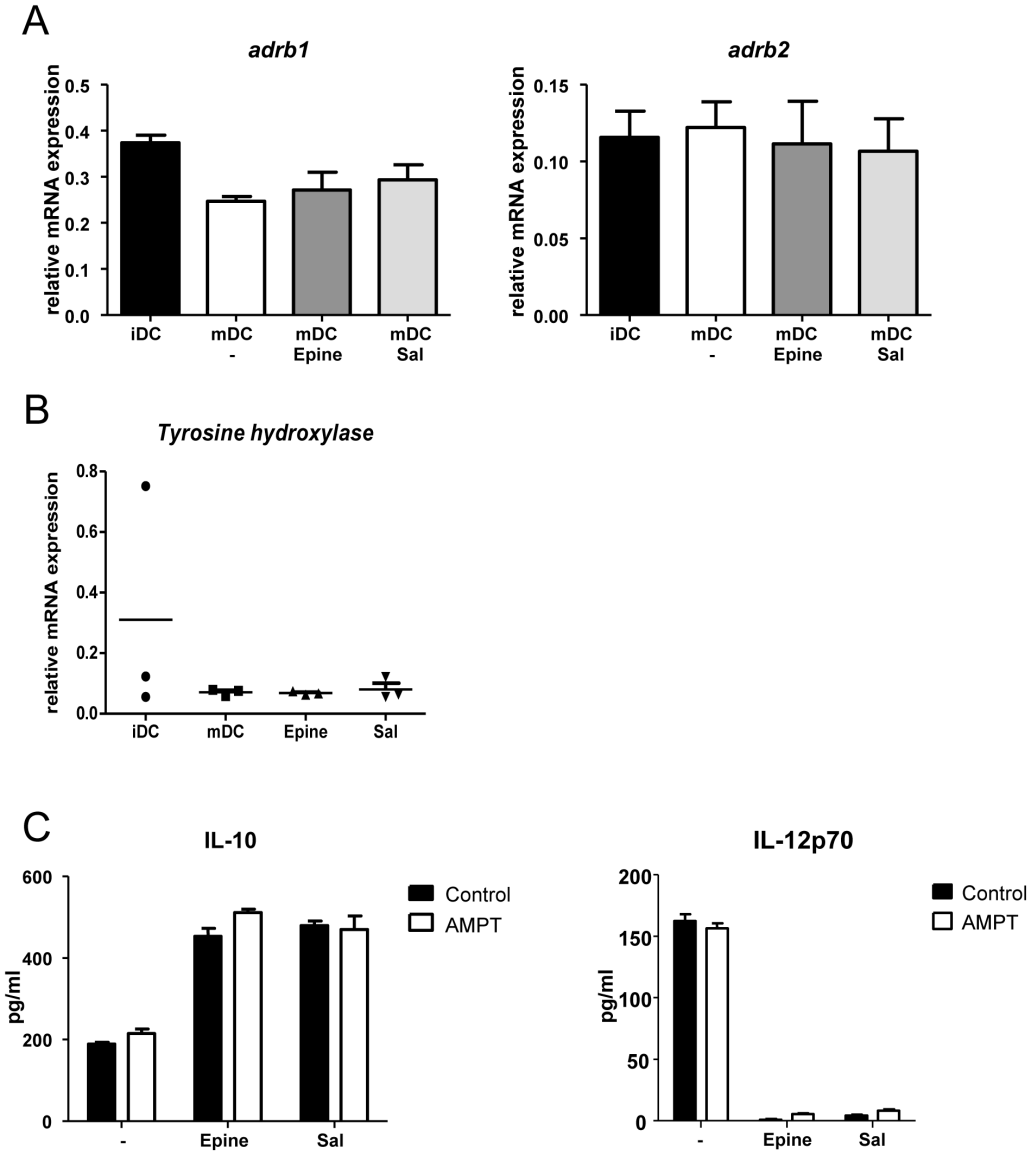
The relative mRNA expression levels of the AR-βe r*adrb1*) and AR-β an*adrb2*) in immature BMDC, and matured BMDC matured in the presence of epinephrine or salbutamol (Panel A). AR-β3 receptors are not expressed in BMDC. Panel B, the expression of the enzyme for catecholamine production, Tyrosine hydroxylase (TH) expression is not affected by epinephrine in matured BMDC. Panel C, blocking of endogenous TH activity by AMPT does not affect the potential of epinephrine or salbutamol to modulate IL-10 and IL-12p70 production. The data represent the mean ± SEM of three independent experiments representative of 5 experiments. * p<0.05, ** p<0.01, ***p<0.001 (ANOVA).

Next, to explain the cytokine changes elicited by adrenergic compounds, we analyzed the expression of adrenergic receptors on maturing BMDCs. We confirmed that BMDCs express the muscarinic receptors M(3), M(4) and M(5) (data not shown). In addition, BMDCs expressed profound transcript levels of *adrb1* and *adrb2* throughout the maturation process (fig. 3A). Likewise, no changes in AR-α subtypes were noted (not shown). Taken together, these results indicate that BMDCs matured in the presence of (nor)epinephrine or salbutamol change their cytokine profile from a pro-inflammatory to an anti-inflammatory repertoire via AR-β2 activation.

BMDCs and macrophages were reported to express the rate-limiting enzyme for catecholamine production, tyrosine hydroxylase (TH) [16]. We analyzed whether TH expression was altered by epinephrine- incubation (fig. 3B), but the transcript levels of TH in maturing BMDCs after pre-exposure to epinephrine showed no different expression pattern. Moreover, blocking the enzymatic activity of TH using α-methyl-DL-tyrosine methyl esther hydrochloride (AMPT) did not affect the AR potential to induce an anti-inflammatory cytokine response in treated BMDCs (fig. 3C). Hence, the effect of AR-β2 activation on BMDC cytokine secretion did not involve the autocrine activity of endogenously produced catecholamines.

### Adrenergic agonists enhance endocytosis in immature BMDCs

**Figure 4:**
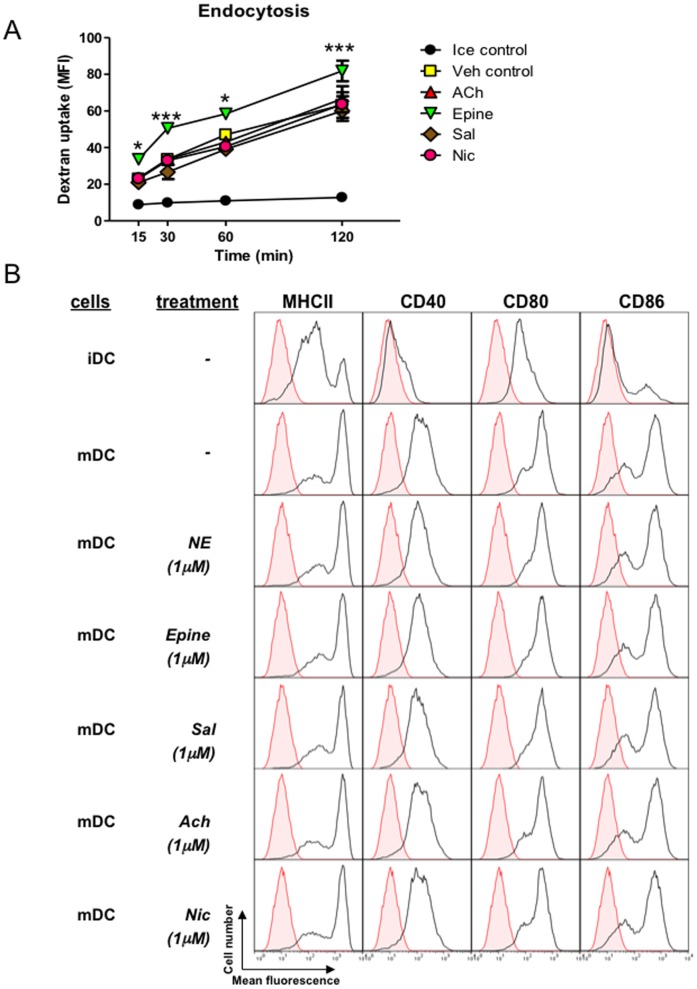
The effects of ACh, epinephrine, salbutamol and nicotine on BMDC endocytosis of FITC-Dextran. Panel A shows a time course of endocytosis in treated BMDCs. CD11c gated immature BMDC endocytosis was assessed using flow cytometry. Results are expressed as mean fluorescence intensity and are the mean ± SEM of three experiments. Statistical significance was calculated using one way ANOVA. * p<0.05, ***p<0.001 versus control (37°C). Panel B: overlay histograms of BMDC stained for MHCII and co-stimulatory molecules CD40, CD80 and CD86 after 24 hours of LPS stimulation were determined by flow cytometry. Cell populations in the grey area indicate the specific isotype control. Both MHCII and co-stimulatory molecules are up-regulated by immature BMDC compared to LPS stimulated matured BMDC. Incubation with the various neurotransmitters before or during maturation has no effect on the levels of maturation markers. Numbers on each histogram indicate the geometric mean fluorescence intensity of cell population for each molecule from representative data of three experimental groups.

As the cytokine response also depends on antigen uptake, we next examined whether the potential of immature BMDCs to take up antigen was affected by pre-incubation with neurotransmitter. As endocytosis is a very important step for antigen presentation by BMDCs, we first examined the effect of adrenergic agonists epinephrine, and salbutamol, and cholinergic agonists ACh, and nicotine, on the endocytotic uptake of inert dextran particles. To this end, immature BMDCs were pre-treated with the respective agonists and allowed to take up FITC-labeled dextran molecules. BMDCs pre-incubated with epinephrine, but not cholinergic agonists, exhibit a higher endocytic activity compared to control BMDCs (fig. 4A). Furthermore, this effect was not mimicked by incubation with salbutamol, suggesting that epinephrine stimulates endocytosis in dendritic cells via α-AR, confirming earlier reports [17]. Pre-treatment of immature BMDCs with ACh, nicotine, or other cholinergic agonists did not lead to a changed uptake compared to control.

We then determined whether the epinephrine treatment of immature BMDCs altered the cellular maturation and the surface expression of MHCII and the co-stimulatory molecules CD80, CD86, CD40. To that end we pre-treated BMDCs with the various neurotransmitters, matured the cells with LPS, and assessed cell surface markers (fig. 4B). As expected, LPS stimulation of BMDCs increased the expression of MHCII and the co-stimulatory molecules CD80, CD86 and CD40 compared to un-stimulated immature cells. Pre-treatment of immature BMDCs with the various neurotransmitters prior to LPS maturation did not modulate the expression levels of MHCII, CD40, CD80 or CD86. These data indicate that neither β-adrenergic nor cholinergic receptor activation alters the maturation process of BMDCs upon LPS challenge.

### Adrenergic agonists imprint BMDCs to stimulate Th2 and regulatory Foxp3^+^ T cell differentiation

**Figure 5:**
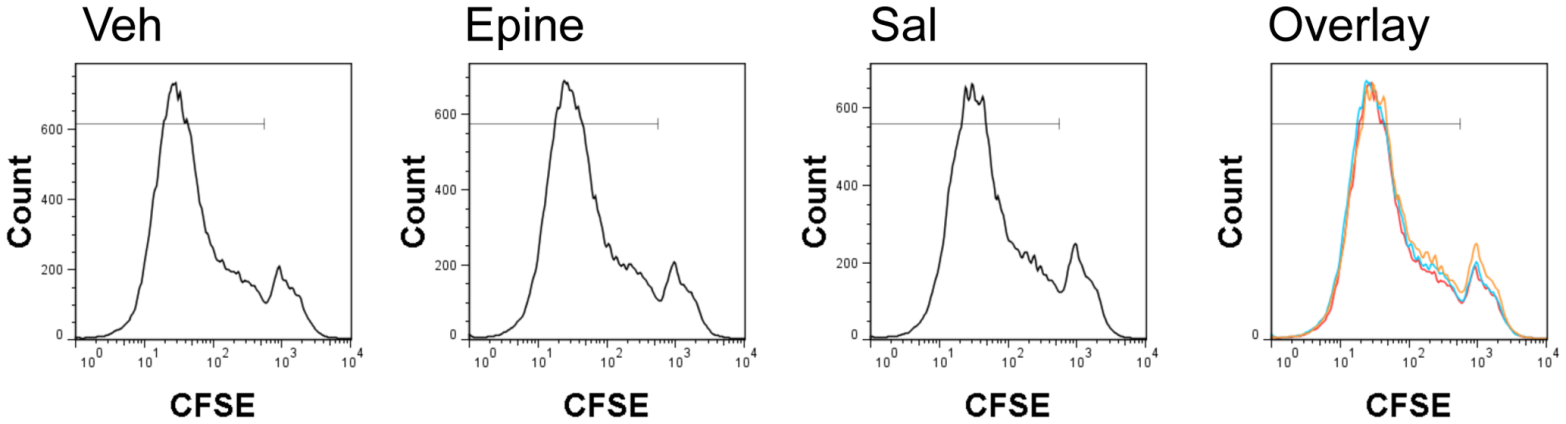
Incubation of vehicle (veh), epinephrine (epine), and salbutamol (sal) of immature Ovalbumin loaded BMDC in a T cell proliferation assay. Cells were incubated during LPS maturation o/n. The cells were washed and freshly isolated naïve CD4 OT-II cells were stained with CFSE. Cells were co-cultured for 3 days and CFSE dilution was determined by flow cytometry. Overlays shown are representative of 3 independent experiments.

Given the potential of AR-activation to alter BMDCs cytokine profiles and antigen uptake we next examined its functional capacity to modulate antigen specific T cell skewing. To this end we primed immature BMDCs with Ovalbumin (OVA) and assessed whether AR-activation changed the polarization of proliferating OVA-specific TCR transgenic (OT-II) T cells. In the first set of experiments, we assessed the effect of AR activation on the capacity of OVA-matured BMDCs to stimulate proliferation of antigen-specific T cells (Fig. 5). As is indicated, a brief stimulation of either epinephrine or AR-β2 selective salbutamol treatment of BMDCs did not change the number of proliferating cells, nor did this treatment alter the rate of T cell division.

**Figure 6:**
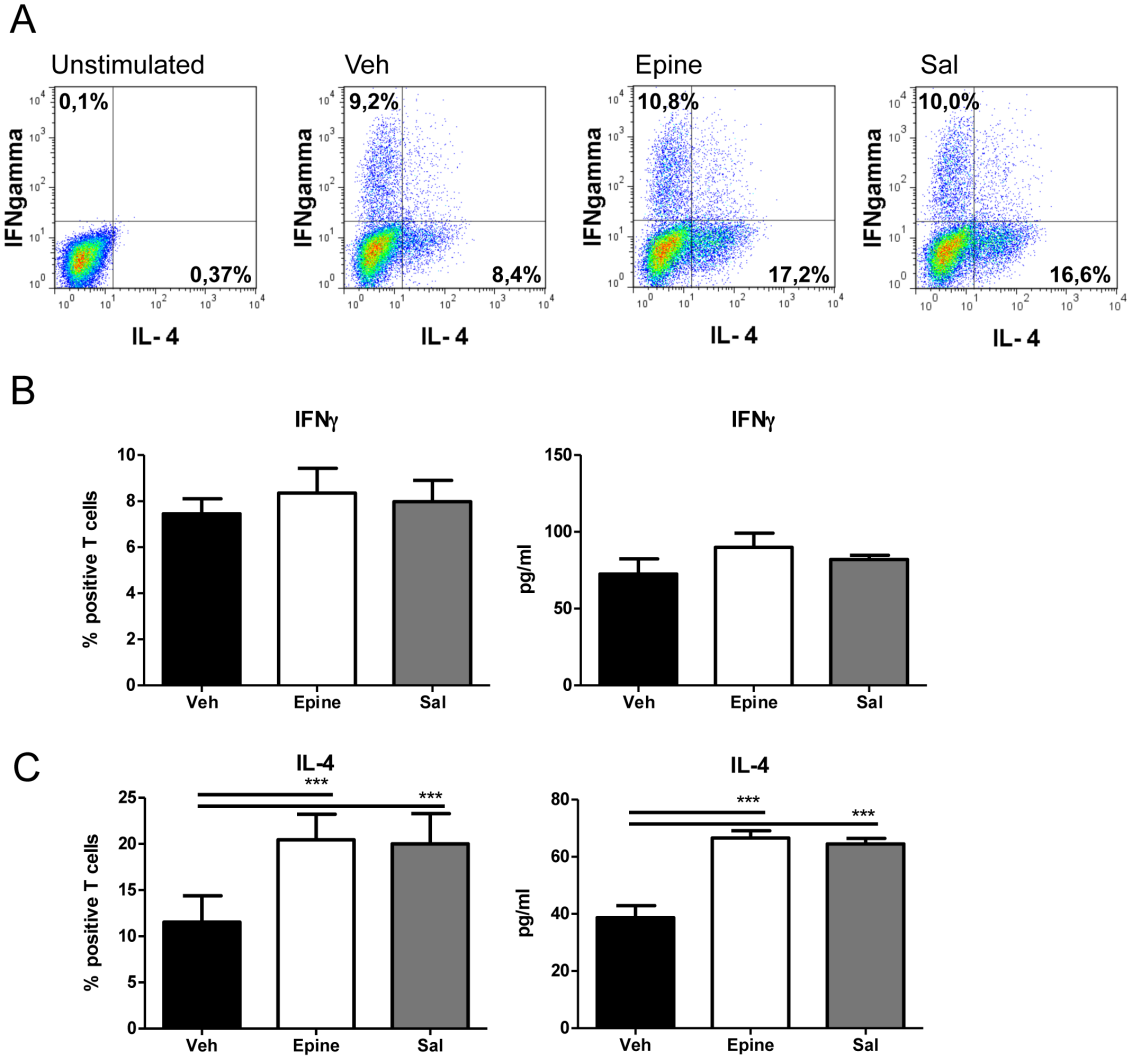
The effect of adrenergic agonists on BMDC potential to skew Tcells. Panel A. FACS plots of intracellular IFNγFand IL-4 in -OT-II T cells co-cultured with BMDC pre-treated with vehicle (Veh), epinephrine (Epine) or salbutamol (Sal) (all at 1 µM). Intracellular IFNγ FNr salarerer affected, while IL-4 production is increased after AR-βaffected, while IL-4 productB, histograms of % IFNγ positive T cells by FACS and by ELISA of day 4 of co-culture supernatant. Panel C, histograms of % IL-4 positive T cells by FACS and by ELISA. Data are expressed as % positive of CD4 gated T cells or as pg/ml and represent the mean ± SEM of three independent experiments representative of 4 experiments. ***p<0.001 (ANOVA).

We examined whether AR-β2 activation would have consequences for the potential of BMDCs to generate particular phenotype differentiation of OVA-specific T cells. Epinephrine or salbutamol pre-exposure to iBMDCs and subsequent maturation with LPS did not significantly affect the potential of mature BMDCs to induce Th1 cells as no enhanced percentage of cells producing IFNγ was detected (fig. 6A–B). Similarly, no difference in intracellular IL-17A (fig. S1), or retinoic acid enzyme activity (fig. S2) was detected. In contrast, epinephrine or salbutamol pre-exposure to iBMDCs led to a significant induction of Th2 cells as determined by enhanced percentage cells having intracellular production of IL-4 (Fig. 6A and C).

**Figure 7:**
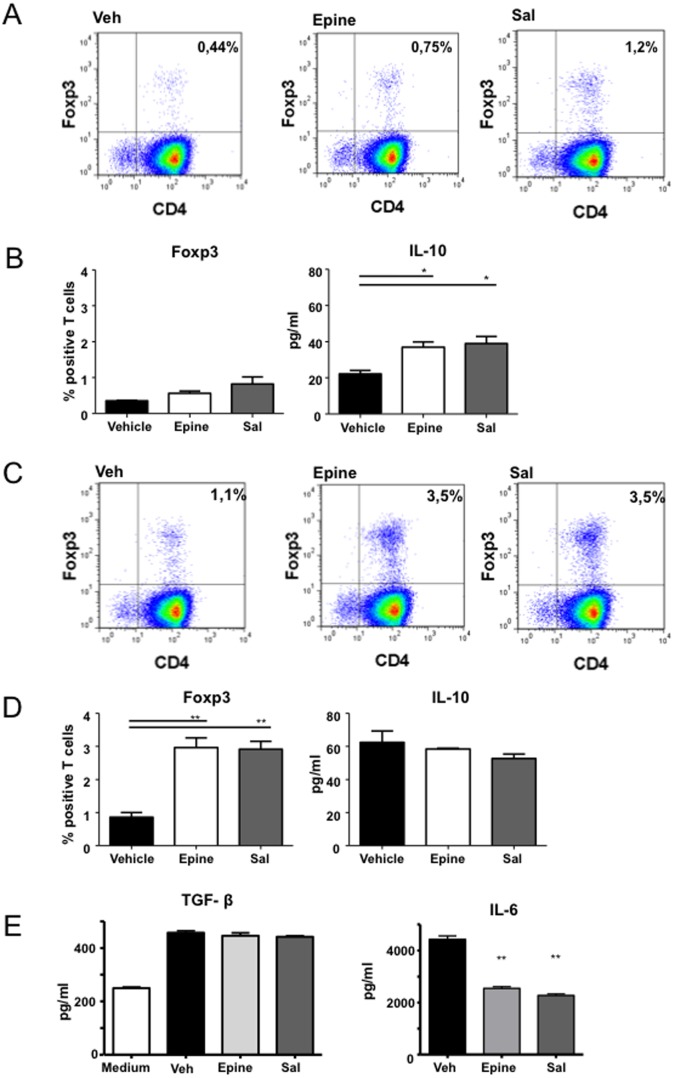
Adrenergic agonists exposure stimulates skewing of a Treg population. Panel A shows flow cytometry analyses of intracellular staining of Foxp3 positive T cells skewed by BMDC treated with indicated AR agonist. Panel B, histograms of Foxp3 positive T cells (left) and IL-10 (right) concentrations in supernatant representative of three independent experiments. Panel C, FACS analyses and, panel D histograms of T cells skewed as described in fig. 7A with added TGF-β, to stimulate Foxp3 differentiation. Panel E shows the concentrations of TGF-β analyses of intracellular staining of Foxp3 posVeh), epinephrine (Epine) and salbutamol (Sal). Data are expressed in % positive of CD4 gated T cells (left) or as pg/ml (right) and represent the mean ± SEM of three independent experiments representative of 4 experiments. *p<0.05 **p<0.01 (ANOVA).

Next to the effect on Th2 induction, a prominent effect of AR-β2 stimulation of DCs was a significant up-regulation of the induction of regulatory T cells that expressed Foxp3 (fig. 7 A–B). We verified that the potential of epinephrine to induce Foxp3-positive T cells was dose-dependent, with the highest activity at 1 µM epinephrine (not shown). Moreover, in the cell supernatant containing this Foxp3 expressing T cell fraction, IL-10 production was significantly increased compared to vehicle treated BMDC T cell co-culture epinephrine (fig. 7 A–B). The potential to induce regulatory T cells was enhanced with the addition of TGF-β to the medium. Under these conditions, epinephrine exposure to the maturing BMDCs synergizes with TGF-β leading to a significant up-regulation of Foxp3 positive T cells compared to culture with TGF-β alone (fig. 7C–D). However, the epinephrine mediated increase in IL-10 secretion was no longer observed with the exogenous addition of TGF-β (fig. 7D). The latter effect is probably due to the previously reported potential of TGF-β (fig. 7D) [18]. Since TGF-β (and retinoic acid-fig. S2) production by BMDCs are not influenced by AR-β2 activation (fig. 7E) the dose dependent increase in differentiation of Foxp3 positive T cells after AR-β2 stimulation could be dependent on IL-6, which significantly decreases after AR-β2 activation (fig. 7E right panel).

## Discussion

In the current study we establish that myeloid DCs respond to adrenergic stimulation by up-regulating their potential to induce T-regs. Our data reveal an important functional effect of the sympathetic system to “imprint” DCs leading to altered cytokine secretion and antigen presenting function. The principle is now generally established that the immune system can no longer be regarded as an autonomous functioning entity but clearly receives regulatory input from neuronal systems. Many neurotransmitters and receptors are shared between the immune system and the nervous system, substantiating claims of a strong regulatory component of the nervous system in immune responses. A good example thereof is the discovery of acetylcholine producing memory T cells in the circulation [19], [20] and spleen [8]. It is likely that such cells can interact with other APCs or feed back on nerve terminals in primary or secondary lymphoid organs.

DCs have been described to express cholinergic as well as AR of several subclasses. Our study shows that DCs express β-ARs, as well as α1 and α2 ARs, as described by others, reviewed by [21]. The ARs are G protein-coupled receptors that are composed of α1 (A,B,C) α2 (A, C, D) and β (β1, β2, and β3) that are coupled with Gq, Gi and Gs proteins respectively. However, as with many other neurotransmitters or their receptors the functional role of AR on APCs is not entirely clear, as functional responses to epinephrine may be dose, time and AR dependent.

Our data imply two important findings: firstly, although DCs respond to general nicotinic cholinergic receptor agonists, there is a strong additional potential of AR to modulate DC APC function, and secondly AR activation on BMDCs stimulates skewing of Th2 and Treg populations. In human monocyte-derived dendritic cells [22] and mouse BMDCs, epinephrine treatment has been described earlier to suppress DC production of proinflammatory cytokines, including IL-12 and TNF-α, whereas it enhances the production of IL-10, similarly to cAMP-elevating agents such as forskolin [17], [21], [23], [24]. However, the functionality of the latter observation is less well documented. We show here a potent immune modulating effect on DCs via expression of their AR-β2, rather than cholinergic receptors, and reveal that the epinephrine activated pathway is a strong stimulant of Treg differentiation. These Tregs generated via (nor)epinephrine conditioned DC may have functional suppressive properties via production of IL-10 *in vitro*, although we cannot ascertain that the Treg fraction is the only cellular source of IL-10 in our system. Epinephrine enhances DC endocytosis within a few minutes via α2-AR, confirming earlier reports [17]. Twenty minutes exposure of DCs with epinephrine (followed by washout) was required before LPS maturation to achieve imprinting of DC antigen presenting function towards an anti-inflammatory phenotype with Treg promoting skewing activity. No effect was seen on the inductive capacity to skew Th17 responses as shown earlier [25], and [26], but this may be due to the use of C57/Bl6 mice cells that favor a Th1 phenotype.

Stimulation of IL-10 production after AR-stimulation of IL-10 production after AR- mice cells that favor a Th1 autocrine effect hindering the production of IL-12p70, TNF-α and IL-6, but we have shown here that blocking IL-10R or neutralizing TGF-β, does not affect the potential of AR-β2 activation to enhance IL-10 secretion, nor the reduction IL-12p70 production. In fact, these effects were mimicked by direct cAMP stimulation using PGE2, confirming earlier data [22]. From this we can conclude that the anti-inflammatory effect of β2-AR activation on maturing DCs is the result of the direct AR-β2 activation and not of the changed cytokine milieu. The effect of epinephrine on cytokine production seems to be mainly mediated via β-ARs coupled with Gs proteins responsible for the elevation of intracellular cAMP levels and the activation of protein kinase A.

Further investigation is needed to unravel the exact underlying mechanism by which epinephrine enhances Treg polarization. Production and release of IL-12p70 by DCs will skew naïve T cells into Th1 IFNγ producing cells, while IL-4 will result in the differentiation of Th2 cells. In mice, interaction between DC and naïve T cells in the presence of TGF-β and IL-6 is required for Th17 induction and IL-23 is important for Th17 expansion [27], [28]. Finally TGF-β in conjunction with the release of RA induces Foxp3 Tregs which are able to actively suppress inflammation via their release of IL-10 [29], [30]. In our study we observed a dose-dependent activity of epinephrine to induce the potential of DCs to give rise to regulatory T cells, an effect that is likely due to the cytokine profile. The reduced levels of IL-6, in conjunction with the modest potential to affect IL-4 release (that reduces IL-10 and TGF-β release by Treg [31]), is a likely explanation for the observed tolarizing effect of epinephrine. Notably, IL-6 serves a critical role in altering the balance between Treg and Th17 cells, stimulating Th17 cell generation along with TGF-β. As we found no change in Th17 nor TGF-β As we found no change in Th17 nor TGF-β generation along with TGF-β altering the balance between Treg and Th17 cells, stimulating tolerogenic potential of treated DCs.

It seems likely that the adrenergic alteration of cytokine balance is involved, at least in part, in immune suppression upon chronic stress. Stress plays an important role in pathogenesis and progression of various diseases [32]. Several stressors induce immune alterations thereby influencing susceptibility or severity of immune disorders such as infection and allergy. Stress activates the sympathetic nervous system and induces (nor)epinephrine secretion. Stress also activates the sympathetic–adrenal–medullary axis and the hypothalamic–pituitary–adrenal axis thereby inducing secretion of epinephrine from the adrenal medulla. As such, catecholamines act as hormones and have a direct impact on the immune responses systemically. In addition, sympathetic nerve fibers also extend to lymphoid organs such as thymus, spleen, lymph nodes, gut-associated lymphoid tissue, and bone marrow [33], [34]. Hence our data imply that (nor)epinephrine released from the ends of sympathetic nerve fibers may represent a strong immunomodulation system via ARs expressed on tissue DCs in lymphoid organs, a system that adds to the already known parasympathetic immunemodulatory properties.

Together, our data show that the DCs functional characteristic as APCs is potently skewed by pre-exposure to AR-β2 agonists. This observation awaits further analyses of different subsets of tissue DCs at different stages of activation may be sensitive to (nor)epinephrine release during stressful episodes. This implies that DCs expressing ARs may be targeted in various stages of inflammatory immune-mediated diseases.

## Supporting Information

Figure S1 **The effect of adrenergic agonists on BMDC Th17 cell skewing.** Panel A, FACS plots of intracellular IL-17A. BMDC pre-treatment with epinephrine or salbutamol does not affect Th17 differentiation. Panel B, histograms of IL-17A positive T cells by FACS (left) and (right) IL-17A concentrations in culture supernatant are not affected. Data are expressed in % positive of CD4 gated T cells (left) or as pg/ml (right) and represent the mean ± SEM of three independent experiments.
(TIFF)

Figure S2 **Aldefluor assay for detection of RALDH activity in T cells.** Panel A, left FACS plots are the control with aldeflour inhibitor DEAB, right FACS plots (test) show active Aldefluor activity of BMDC. There is no effect of epinephrine on RALDH enzyme activity. Panel B, left, histogram of mean fluorescence intensity of FACS plots. Right histogram shows percentage (%) of BMDC positive for RALDH activity. Data show means ± SEM of 3 independent experiments.
(TIFF)
