# Supplementary figures and images for: Adrenergic β2 Receptor Activation Stimulates Anti-Inflammatory Properties of Dendritic Cells In Vitro
Source: PLoS One. 2014 Jan 22;9(1):e85086. doi: 10.1371/journal.pone.0085086 (PMC3898911; doi:10.1371/journal.pone.0085086)

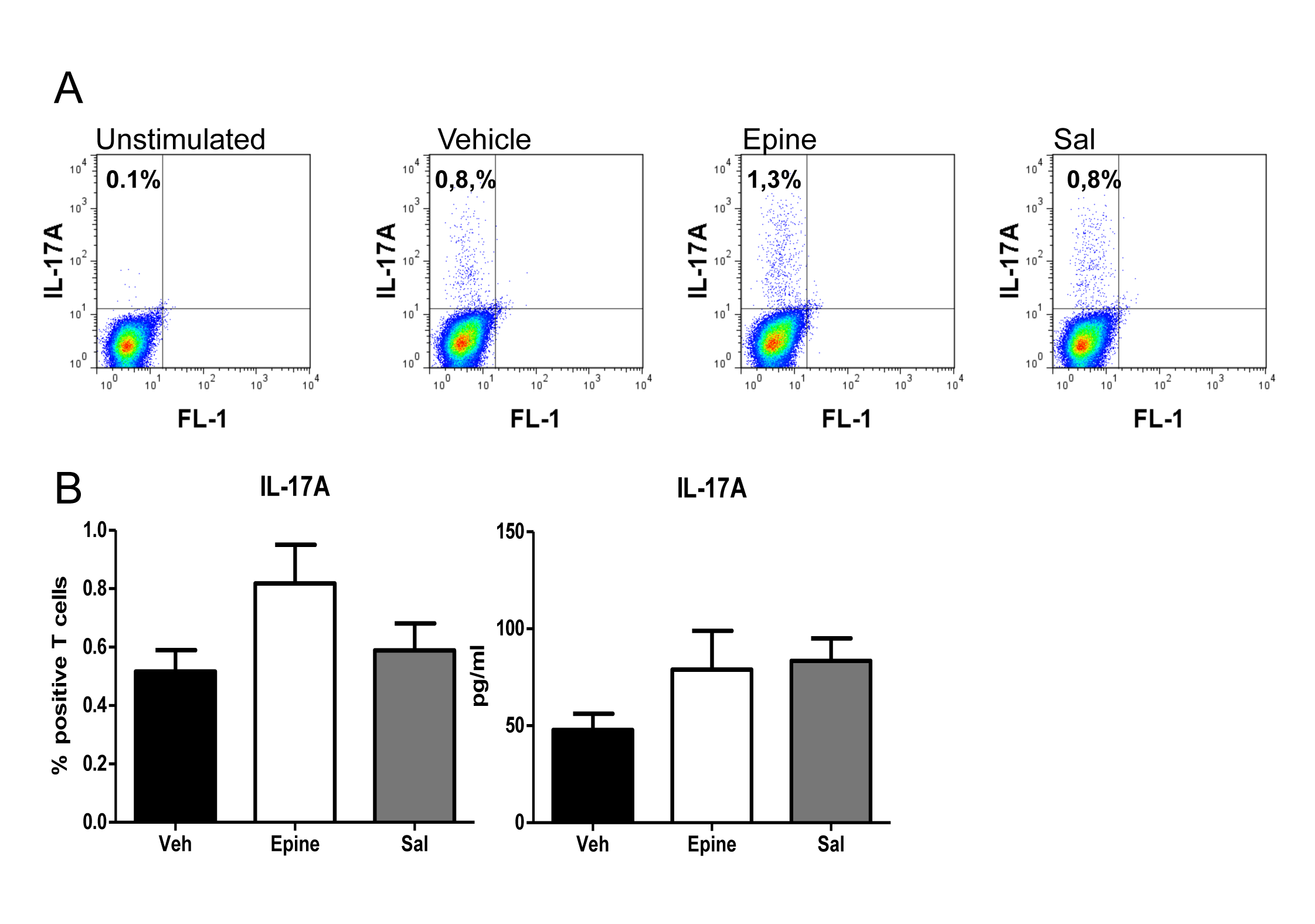

Supplement: Figure S1 — The effect of adrenergic agonists on BMDC Th17 cell skewing. Panel A, FACS plots of intracellular IL-17A. BMDC pre-treatment with epinephrine or salbutamol does not affect Th17 differentiation. Panel B, histograms of IL-17A positive T cells by FACS (left) and (right) IL-17A concentrations in culture supernatant are not affected. Data are expressed in % positive of CD4 gated T cells (left) or as pg/ml (right) and represent the mean ± SEM of three independent experiments. (TIFF) [file pone.0085086.s001.tiff]

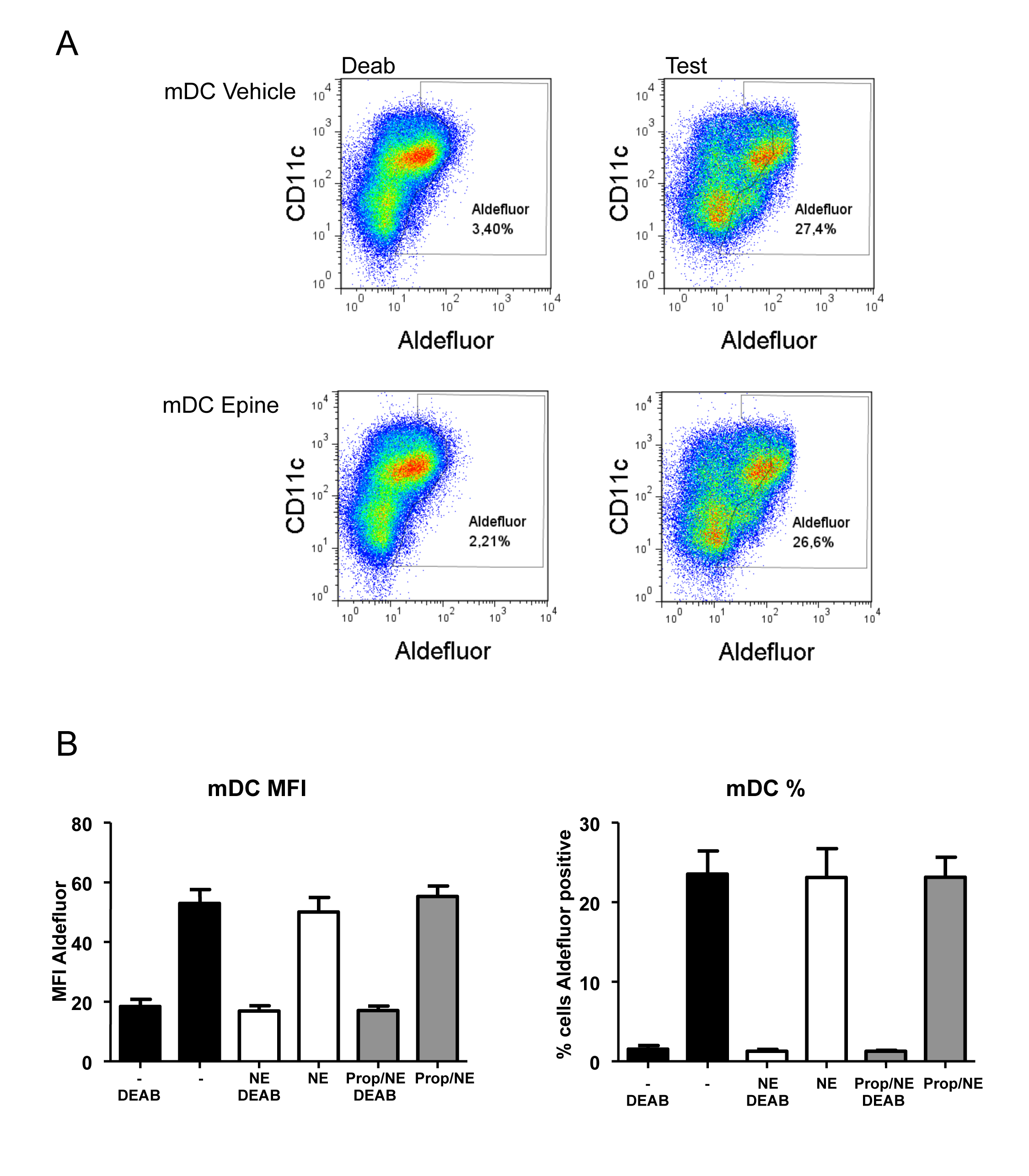

Supplement: Figure S2 — Aldefluor assay for detection of RALDH activity in T cells. Panel A, left FACS plots are the control with aldeflour inhibitor DEAB, right FACS plots (test) show active Aldefluor activity of BMDC. There is no effect of epinephrine on RALDH enzyme activity. Panel B, left, histogram of mean fluorescence intensity of FACS plots. Right histogram shows percentage (%) of BMDC positive for RALDH activity. Data show means ± SEM of 3 independent experiments. (TIFF) [file pone.0085086.s002.tiff]
